# Pills of Multi-Target H_2_S Donating Molecules for Complex Diseases

**DOI:** 10.3390/ijms25137014

**Published:** 2024-06-27

**Authors:** Angela Corvino, Antonia Scognamiglio, Ferdinando Fiorino, Elisa Perissutti, Vincenzo Santagada, Giuseppe Caliendo, Beatrice Severino

**Affiliations:** Department of Pharmacy, School of Medicine, University of Naples Federico II, Via D. Montesano 49, 80131 Napoli, Italy; antonia.scognamiglio@unina.it (A.S.); fefiorin@unina.it (F.F.); perissut@unina.it (E.P.); santagad@unina.it (V.S.); caliendo@unina.it (G.C.); bseverin@unina.it (B.S.)

**Keywords:** multi-target directed ligand, hydrogen sulfide, H_2_S donors, multi-target compounds, molecular hybridization

## Abstract

Among the various drug discovery methods, a very promising modern approach consists in designing multi-target-directed ligands (MTDLs) able to modulate multiple targets of interest, including the pathways where hydrogen sulfide (H_2_S) is involved. By incorporating an H_2_S donor moiety into a native drug, researchers have been able to simultaneously target multiple therapeutic pathways, resulting in improved treatment outcomes. This review gives the reader some pills of successful multi-target H_2_S-donating molecules as worthwhile tools to combat the multifactorial nature of complex disorders, such as inflammatory-based diseases and cancer, as well as cardiovascular, metabolic, and neurodegenerative disorders.

## 1. Introduction

In the latter decades of the twentieth century, the doctrine of “one drug–one target–one disease” philosophy was the only approach to characterize the research in the field of medicinal chemistry. Despite the best efforts of medicinal chemists, this strategy resulted to be inadequately in many diseases because of their multifactorial nature. 

Recently, multi-target drug discovery strategy has emerged as promising alternative to the classical design approach [1,2].

This strategy is based on molecular hybridization, which consists in combining two pharmacologically active compounds, or parts of them (fragment-based), in a single chemical entity. This new molecule, a multi-target directed ligand (MTDL), derived from framework combination, connected directly via a metabolically stable or cleavable linker, or framework integration strategies. Depending on the degree of the integration, the MTDL can be fused or merged.

Several studies confirmed that MTDLs were responsible for a synergic therapeutic effect and reduced adverse side effects, compared to modulation of a single target [3,4,5,6]. These improvements justify the growing interest of the medicinal chemistry community in MTDLs. 

Considering the success in this field, in this review we present the application of a multi-target drug approach involving the use of hydrogen sulfide (H_2_S) donor units for multifactorial disorders. 

**Figure 1:**
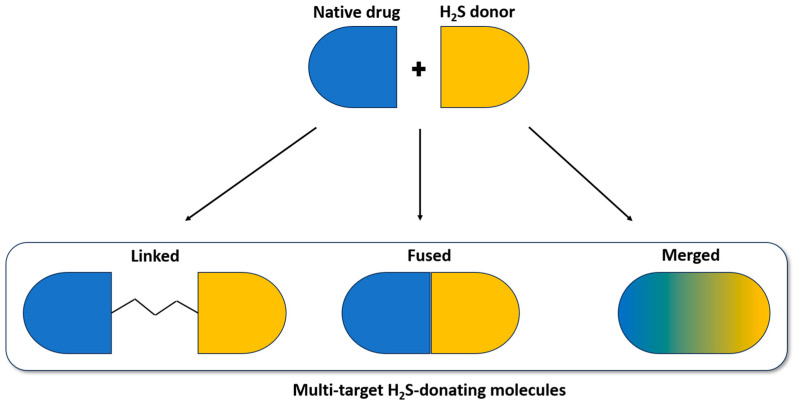
Multi-target drug design strategy of H_2_S donating molecules based on framework combination. The two moieties, native drug (in blue) and H_2_S donor (in yellow), can be connected via metabolically stable or cleavable linkers, attached directly (fused) or merged.

Herein, we focused on the development of multi-target H_2_S-donating molecules obtained by combining two chemical entities, a native drug with a moiety structurally able to release H_2_S, either merged or connected directly (fused) or via a linker (Figure 1).

Through the application of this strategy, new molecules capable of simultaneously targeting multiple therapeutic pathways, resulting in improved treatment outcomes, are obtained.

Some of these hybrid compounds with an interesting activity profile against different targets have the potential to be developed as drug candidates for the treatment of several complex diseases.

## 2. H_2_S: From Historical Background to Developing Chemical Tools

H_2_S has long been known as the third gasotransmitter, along with nitric oxide (NO) and carbon monoxide (CO). Among this family, H_2_S has the uniqueness of existing in multiple forms in nature: gas, solid as salt, or liquid as an aqueous solution establishing a dynamic equilibrium among molecular hydrogen sulfide and ionized forms (sulfide and bisulfide ions) under physiological conditions.

Although the existence of H_2_S in mammalian tissue has been recognized for decades, its endogenous production and signaling potential were not fully understood until the seminal study by Abe and Kimura in 1996 [7].

In mammals, H_2_S is primarily produced by cystathionine-β-synthase (CBS), cystathionine-γ-lyase (CSE), and 3-mercaptopyruvate sulfurtransferase (3-MST)/cysteine aminotransferase (CAT) [8,9]. 

Despite being traditionally considered a toxic gas [10,11], over the last decade, significant research and development efforts have been focused on H_2_S in order to comprehend its biologic roles in health and disease and its positive role in crucial physiological functions.

It is widely considered a key mediator in many physio-pathological processes, such as inflammation, neuromodulation, oxidation, tumor progression, cardiovascular, bone, and metabolic disease [12,13].

As interest in the physio-pathological aspects of H_2_S has expanded, chemical tools for elucidating the role of H_2_S have been developed. For this purpose, multiple approaches have been identified, either based on inhibition of H_2_S biosynthesis or H_2_S donation.

Currently, a limited number of pharmacologically well-characterized compounds are considered to be selective and potent inhibitors of the enzymes involved in H_2_S biosynthesis. Specifically, DL-propargylglycine, β-cyanoalanine, L-2-oxo-N-(prop-2-yn-1-yl) thiazolidine-4-carboxamide, aminooxyacetic acid, and L-aspartic acid (L-Asp) are the most selective agents to block the H_2_S endogenous production [14,15,16].

Additionally, the use of exogenous H_2_S in different disease models has been extensively studied to further explore the plethora of biological effects of this gaseous signaling molecule.

To date, available H_2_S donor sources are limited. The widely used pharmacological tools are inorganic sulfide salts, such as sodium sulfide (Na_2_S) and hydrosulfide (NaHS), that quickly release H_2_S upon reaction with water [17].

**Figure 2:**
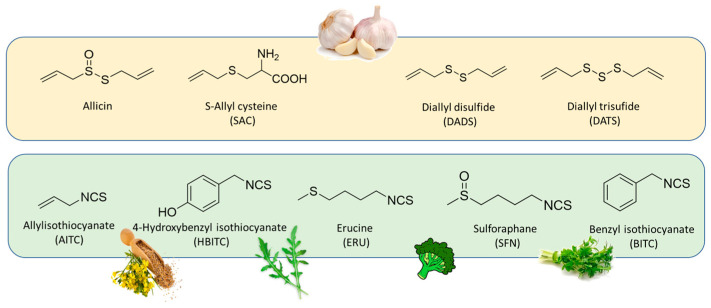
Chemical structures of natural H_2_S donating compounds: garlic-derived organosulfur compounds (yellow box) and natural isothiocyanates from *Brassicaceae* family (green box).

Among the natural sources, interesting candidates as H_2_S-donor were represented by garlic-derived organosulfur compounds, such as allicin and the polysulfides diallyl di and tri-sulfide (DADS and DATS), and isothiocyanates, such as sulforaphane (SFN), allyl isothiocyanate (AITC), benzyl isothiocyanate (BITC), 4-hydroxybenzyl isothiocyanate (HBITC), and erucin (ERU), present in many edible plants of the *Brassicaceae* family, like broccoli, black and white mustard, garden cress, and rocket (Figure 2).

Among the synthetic H_2_S donors, the most widely investigated is the 4-methoxyphenyl(morpholino)phosphinodithioate morpholinium salt, GYY4137 [18]. This compound is one of the first slow-releasing H_2_S donors developed that decomposes spontaneously in aqueous buffers to release H_2_S over a long period of time. 

**Figure 3:**
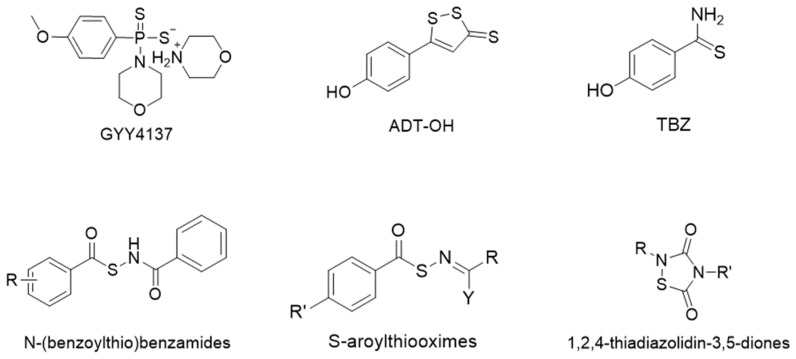
Chemical structures of most representative H_2_S donors.

Moreover, other H_2_S-releasing compounds were developed and investigated, including 1,2-dithiole-3-thiones, such as 5-(4-hydroxyphenyl)-3H-1,2-dithiole-3-thione (ADT-OH) [19], arylthioamides, such as 4-hydroxythiobenzamide (TBZ) [20], N-(benzoylthio)benzamides [21], S-aroylthiooximes [22], 1,2,4-thiadiazolidin-3,5-diones [23], dithioates, such as 4-hydroxybenzodithioate (HBTA) [24], and isothiocyanates [25,26] (Figure 3). 

**Figure 4:**
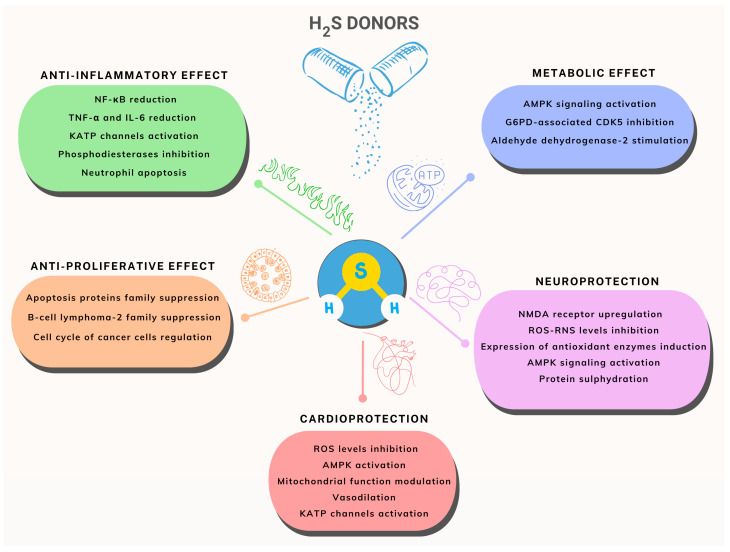
Biological effects and pathophysiological mechanisms of H_2_S donors.

H_2_S donors are known to play their biological roles [27] through several mechanisms, summarized in Figure 4. 

Specifically, H_2_S donors have important benefits in terms of anti-inflammatory and anti-cancer properties through a variety of complex processes. 

First, in terms of anti-inflammatory properties, H_2_S donors reduce inflammation by blocking the production and release of inflammatory mediators and altering inflammatory signaling pathways. In particular, they have the ability to decrease the action of important molecules that regulate inflammation, such as nuclear factor-κB (NF-κB) and tumor necrosis factor-α (TNF-α), as well as reduce the infiltration of inflammatory cells and the levels of inflammatory mediators [28].

In terms of anticancer effects, H_2_S donors have a strong inhibitory impact on tumor development and metastasis by interfering with cancer cells’ biological activities. This includes the modulation of cancer cell survival signaling pathways, such as the suppression of the inhibitor of apoptosis proteins (IAPs) family and the B-cell lymphoma-2 (Bcl-2) family, which induce apoptosis in tumor cells. Furthermore, H_2_S donors have the ability to regulate the cell cycle of cancer cells, preventing their uncontrolled multiplication [29,30].

Moreover, H_2_S donors produce a variety of cardiovascular effects. First, they contribute to blood pressure stability by modulating vascular tension and cellular signaling pathways. Furthermore, research suggests that H_2_S donors may help prevent atherosclerosis by reducing artery wall damage and plaque development [31,32].

H_2_S donors are also therapeutically useful in the treatment of metabolic disorders by controlling lipid metabolism, energy metabolism, insulin signaling, and other processes. In the prospective therapy of metabolic illnesses, H_2_S donors’ function through a variety of channels, positively influencing metabolic processes. Furthermore, H_2_S donors have a considerable effect on lipid metabolism. They can control fatty acid synthesis and breakdown, increase lipid balance, and limit aberrant lipid buildup, all of which contribute to the prevention of metabolic illnesses, including obesity. Particularly, by blocking glucose-6-phosphate dehydrogenase (G6PD)-associated cyclin-dependent kinase 5 (CDK5), stimulating aldehyde dehydrogenase-2 (ALDH2), boosting mitochondrial antioxidant defense, and initiating the adenosine monophosphate-activated protein kinase (AMPK) signaling pathway, H_2_S donors also contribute to glucose metabolism. Finally, these mechanisms protect the function of pancreatic insulin-producing cells (β cells) from damage caused by high glucose [33].

Moreover, prior research has mostly concentrated on the advantages of using exogenous H_2_S for neurodegenerative illnesses. Research has demonstrated that H_2_S is involved in many physiological processes in the body and that, because of its antioxidant qualities and capacity to control oxygen consumption, it can have cytoprotective effects. H_2_S influences the activity of particular proteins by sulfhydrylation or by up- or down-regulating the genes related to anti-inflammatory, antiapoptotic, and antioxidant defenses [34].

## 3. Multi-Target H_2_S Donors

Based on the growing interest in the H_2_S field and the emerging multitarget-directed ligands approach, some of the H_2_S-releasing moieties have been largely used for developing novel molecular hybrids with several “native” drugs [35,36], including non-steroidal anti-inflammatory drugs (NSAIDs), corticosteroids, nucleosides, prostaglandin analogs, adrenergic agonists or antagonists, carbonic anhydrase inhibitors, opioid receptor agonist, acetylcholinesterase (AChE) inhibitor, N-methyl-D-aspartate (NMDA) receptor antagonist, anthracyclines and Transient Receptor Potential Vanilloid 1 (TRPV1) agonists. 

In this review, we focus on the “smart” multi-target compounds resulting from the combinations of “old” native drugs with moieties structurally able to release H_2_S and their applications as therapeutic tools in complex disorders, such as inflammatory-based diseases, cancer, as well as neurodegenerative, cardiovascular, and metabolic diseases [31,34,37,38,39]. 

### 3.1. Pills of H_2_S-Donating Molecules for Inflammatory-Based Diseases

The substantial evidence supporting the involvement of H_2_S in inflammatory processes [28] has prompted scientists to develop novel compounds combining H_2_S-donor moieties with anti-inflammatory drugs, non-steroidal or steroidal compounds, offering a potential new approach for treating inflammation and inflammatory conditions. 

#### 3.1.1. H_2_S-Donating NSAIDs 

NSAIDs have long been used for their anti-inflammatory and analgesic properties. However, they are associated with certain adverse effects, particularly gastrointestinal damage and cardiovascular risks. 

By combining NSAIDs with H_2_S donors, researchers aim to enhance the anti-inflammatory effects of these drugs while simultaneously exploiting the beneficial properties of H_2_S. The hybridization approach offers a dual mechanism of action, potentially resulting in superior therapeutic outcomes and reduced side effects. The incorporation of H_2_S donors into NSAIDs holds great potential in mitigating the side effects of NSAIDs while enhancing the therapeutic efficacy of these drugs. 

The H_2_S released by these hybrid compounds acts as a vasodilator, protecting the gastrointestinal lining and reducing the risk of NSAID-induced gastric ulcers and bleeding. Additionally, H_2_S modulates the production of inflammatory mediators, such as cytokines and prostaglandins, thereby further reducing inflammation and pain. 

**Figure 5:**
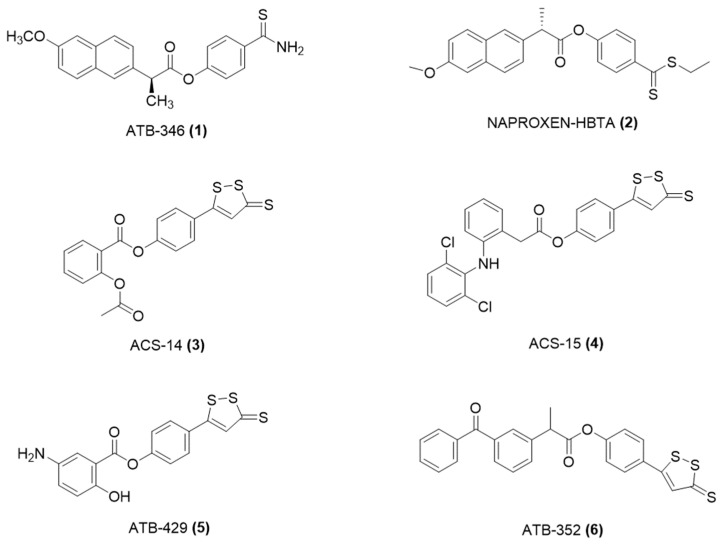
Chemical structures of representative H_2_S-NSAIDs.

One of the most used NSAIDs, naproxen, was modified with a moiety able to release H_2_S, giving ATB-346 [2-(6-methoxy-napthalen-2-yl)-propionic acid 4-thiocarbamoyl-phenyl ester] (Figure 5) [40]. 

The new compound derived from the conjunction of naproxen with TBZ, leading to ATB-346, which not only retains the effects of naproxen but also improves bone quality and prevents gastric mucosa damage due to prostaglandin inhibition [41].

The efficacy of ATB-346 was assessed in healthy subjects across various models with weakened mucosal protection and within a gastric ulcer recovery model. Regarding gastric harm, ATB-346 proved to be roughly 100 times less risky than naproxen in healthy subjects, while also demonstrating effects that were either on par with or more effective than those of naproxen in two inflammation models. Moreover, unlike selective cyclooxygenase-2 (COX-2) inhibitors, ATB-346 did not cause notable gastric injury in rats with impaired mucosal defense; it further promoted the repair of existing gastric ulcers. Significantly, ATB-346 displayed a more favorable cardiovascular profile compared to traditional NSAIDs. It has also been found to inhibit alveolar bone loss and inflammation in models of periodontitis [41].

Additionally, more recent studies have shown that ATB-346 can reduce intestinal inflammation and restore transit in conditions like postoperative ileus [42]. This makes ATB-346 a potentially new adjuvant therapy for periodontal diseases and other inflammatory conditions where NSAIDs are indicated but their side effects are a concern.

More recently, a new H_2_S-releasing naproxen, Naproxen-HBTA, was crafted [24]. It exhibited promise in diminishing the characteristics associated with metastatic melanoma, as observed in animal studies. Research has revealed that naproxen-HBTA can trigger cell death and reduce the progression, invasion, and cluster development of human melanoma cells. Moreover, when administered orally, it markedly reduced the advancement and expansion of melanoma in mouse models.

Another H_2_S-releasing NSAID derivative is ACS14 [2-acetyloxybenzoic acid 4-(3-thioxo-3H-1, 2-dithiol-5-yl) phenyl ester] (Figure 5), a conjugate between ADT-OH and acetylsalicylic acid [43]. This new entity, ACS14, combines aspirin’s anti-inflammatory properties with the protective effect of H_2_S, inhibiting COX and showing antioxidant effects. It stimulated antioxidants, protecting against aspirin-induced gastric damage [43].

Moreover, ACS14, not only blocked the aggregation dependent on arachidonic acid but also, in contrast to regular aspirin, reduced aggregation induced by adenosine diphosphate (ADP), collagen, and thrombin. These effects, which are independent of COX, were noted following both a brief period of whole blood incubation in vitro and sustained oral administration in mice. As a result, ACS14 extended the clotting time, indicative of the rate at which a firm clot develops, a process highly reliant on platelet activity. This is in line with the observed in vivo reduction of arterial thrombus formation in both small arterioles and larger arteries. Additionally, it has been demonstrated that the diminished activation of the αIIbβ3 integrin by ACS14, along with an increase in intracellular cyclic nucleotides, plays a role in its antithrombotic properties. Moreover, it has been demonstrated the inhibitory effects of ACS14 in estrogen receptor-negative breast cancer cells and leukemic Jurkat cells [44,45].

Sustaining its thromboxane-suppressing activity, the aspirin-H_2_S releasing hybrid maintained the integrity of the gastric mucosa by enhancing H_2_S/glutathione (GSH) formation [46]; this process influences redox imbalance. Additionally, a recent study found that ACS14 could protect the gastric mucosa from aspirin-induced damage by inhibiting oxidative stress and stimulating local blood flow, possibly involving ATP-sensitive potassium (KATP) channels [47]. 

ATB-429, a compound derived from the combination of mesalamine and ADT-OH (Figure 5), exhibited enhanced properties for reducing inflammation and pain [48]. Compared to mesalamine alone, ATB-429 has shown a notable decrease in gastrointestinal adverse effects, particularly evident in a colitis mouse model where it demonstrated superior anti-inflammatory capabilities. The compound was effective in mitigating inflammation in conditions such as liver and lung injuries caused by lipopolysaccharides (LPS), as well as in ulcerative colitis. It also offered anti-inflammatory benefits in the case of gastric mucosal damage induced by NSAIDs. ATB-429 outperformed mesalamine in lessening mucosal harm and the severity of the disease; it also significantly diminished the infiltration of chronic granulocytes and lowered the levels of various key inflammatory cytokines’ mRNA. 

ACS15 is a derivative that donates H_2_S, created by combining diclofenac with ADT-OH (Figure 5). It offered superior anti-inflammatory benefits and fewer gastrointestinal side effects compared to diclofenac. ACS15 showed the capability to release H_2_S both in vitro and in vivo, enhancing its anti-inflammatory properties and markedly diminishing lung damage linked to pancreatitis [49]. Additionally, research indicated that ACS15 possesses activity against myocardial ischemia-reperfusion injury, a property not observed in diclofenac [50].

ATB-352 was obtained by fusing ketoprofen with ADT-OH (Figure 5). Studies have shown that ATB-352 exhibited anti-inflammatory effects comparable to ketoprofen while causing negligible gastrointestinal side effects. Moreover, the hybrid had potential for the chemoprevention of tumors [51].

Furthermore, hybrids of NSAIDs and H_2_S and NO donors have also been developed. This approach also aims to improve the effectiveness of the native drugs and minimize their adverse effects.

**Figure 6:**
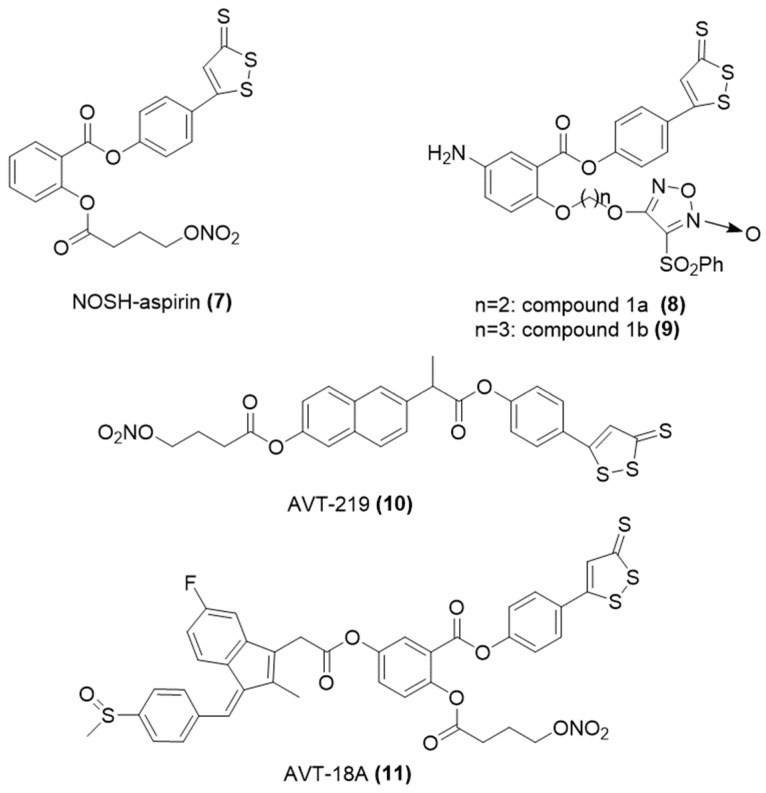
Chemical structures of representative NOSH-NSAIDs.

NOSH-aspirin is a hybrid of aspirin bearing both H_2_S and nitric oxide-releasing entities (Figure 6) [52,53]. The new compound has been shown to maintain the fever-reducing, pain-relieving, inflammation-diminishing, and platelet aggregation-inhibiting properties of aspirin. Conversely, it has been demonstrated to have a reduced risk of gastrointestinal hemorrhage and offer enhanced efficacy in preventing tumors. 

Moreover, Wang and colleagues developed a range of ATB-429 derivatives that release nitric oxide and assessed their capacity to inhibit tumor growth [54]. The findings indicated that these derivatives are potent in combating tumor cells. Specifically, compounds 8 (Figure 6) with an IC_50_ value of 2.677 µM, and 9 (Figure 6) with an IC_50_ of 3.051 µM, were more effective against the MCF-7 breast cancer cell line, and compound 8 (Figure 6) was also more effective (IC_50_ = 1.270 µM) against the DU145 prostate cancer cell line, compared to Vandetanib, which had IC_50_ values of 3.536 µM and 1.974 µM, respectively.

Similarly, AVT-219 and AVT-18A (Figure 6) are NOSH NSAIDs created by fusing naproxen and sulindac, respectively, with H_2_S and nitric oxide donors [55]. Both compounds preserved the anti-inflammatory and anti-platelet aggregation benefits of their native drugs. Moreover, they exhibited a reduced impact on the gastrointestinal tract. Additionally, these NOSH compounds demonstrated potent efficacy in suppressing the proliferation of various cancer cell types, such as colon, breast, and pancreatic cancer cells [56].

#### 3.1.2. H_2_S-Donating Glucocorticoids

Glucocorticoids, a class of corticosteroids, are well-known for their potent anti-inflammatory and immunosuppressive properties. In fact, they represent the standard gold treatment of various inflammatory-based diseases, although their long-term use can lead to adverse effects, including cardiovascular diseases, osteoporosis, and weakened immunity [57,58]. 

To harness the therapeutic benefits of H_2_S in controlling the inflammatory and pruritogenic response while mitigating the side effects of glucocorticoids, researchers have developed H_2_S-donating glucocorticoids. 

H_2_S has shown promising results in treating asthma by activating potassium channels in bronchial smooth muscle cells, leading to relaxation, and by decreasing eosinophil infiltration and oxidative stress in the lungs [59,60]. 

Similarly, in the skin, H_2_S plays a key role in controlling critical processes, including vasodilation, the formation of new blood vessels, the growth and division of cells, programmed cell death, and inflammatory responses [61].

Starting from these findings, a novel therapeutic strategy has been offered particularly in conditions like asthma and atopic dermatitis, where both inflammation and oxidative stress play a significant role.

**Figure 7:**
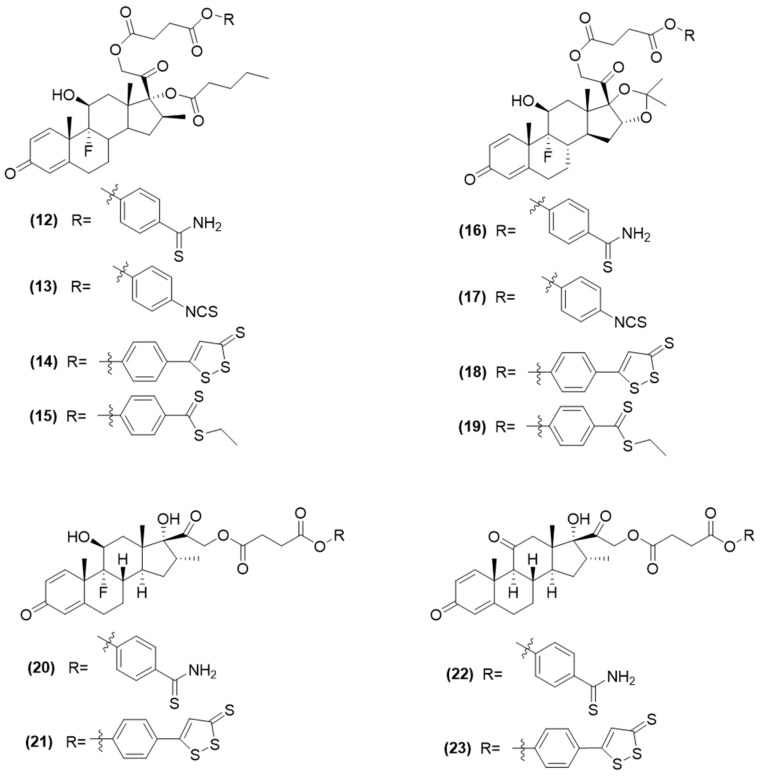
Structures of H_2_S-donating prednisone, dexamethasone, betamethasone and triamcinolone derivatives.

Researchers conjugated different glucocorticoids, such as betamethasone 17-valerate, triamcinolone acetonide, dexamethasone, and prednisone, to various H_2_S-releasing groups, like ADT-OH, thioamides, isothiocyanates, and benzodithioates, using a specific spacer (Figure 7) [62,63]. 

All hybrids, compound 12-23 (Figure 7) have been proven to release H_2_S in both buffer solutions (with thiol activation) and bronchial smooth muscle cells, as confirmed by H_2_S electrode measurements and fluorescent probes [62,63].

Among the synthesized compounds, compounds 13 and 17 [62], and compound 22 [63] (Figure 7) demonstrated the most potent effects and were selected for further investigation. These hybrids exhibited a significantly stronger ability to inhibit mast cell degranulation compared to their parent glucocorticoid. This enhanced anti-inflammatory effect is likely due to the release of H_2_S, as compounds that donate H_2_S, such as 4-hydroxy-phenylisothiocyanate (HPI) and TBZ, also showed strong inhibitory effects.

Notably, compound 22 (Figure 7) exhibited in animal studies protective properties against airway remodeling caused by asthma and significantly reduced the density of smooth muscle and collagen around the bronchioles. It also effectively decreased the infiltration of eosinophils and mast cells in the lung tissue of mice [63,64]. 

More recently, considering the potential benefits of combining H_2_S donors with glucocorticoids and the primary approach to treating various skin disorders, including atopic dermatitis, which involves the application of corticosteroids such as dexamethasone and two H_2_S-releasing derivatives, compounds 20 and 21 (Figure 7), were selected and evaluated in a mouse model of atopic dermatitis [65]. 

Applying equal doses of dexamethasone or its derivatives, compounds 20 and 21, topically led to comparable decreases in dermatitis severity, scratching, swelling, eosinophil count, spleen enlargement, and tissue alterations. Unlike dexamethasone, the H_2_S-releasing hybrids inhibited the rise of IL-4 and the oxidative damage to skin proteins. Specifically, compound 20 and not compound 21, enhanced the H_2_S production and glutathione peroxidase (GPx) enzyme activity when given in equal molar amounts [65].

This study once again demonstrated the efficacy of the combination of H_2_S-donating moiety with dexamethasone, maintaining its anti-inflammatory properties and contributing additional therapeutic benefits to the original drug.

### 3.2. Pills of H_2_S-Donating Molecules as Anti-Cancer Agents

Numerous studies have highlighted the significant role of H_2_S within cancer biology, and, intriguingly, the administration of H_2_S-donating compounds has been linked to the induction of apoptosis in different types of cancer cells [37]. 

#### 3.2.1. H_2_S-Donating Doxorubicin Derivatives

**Figure 8:**
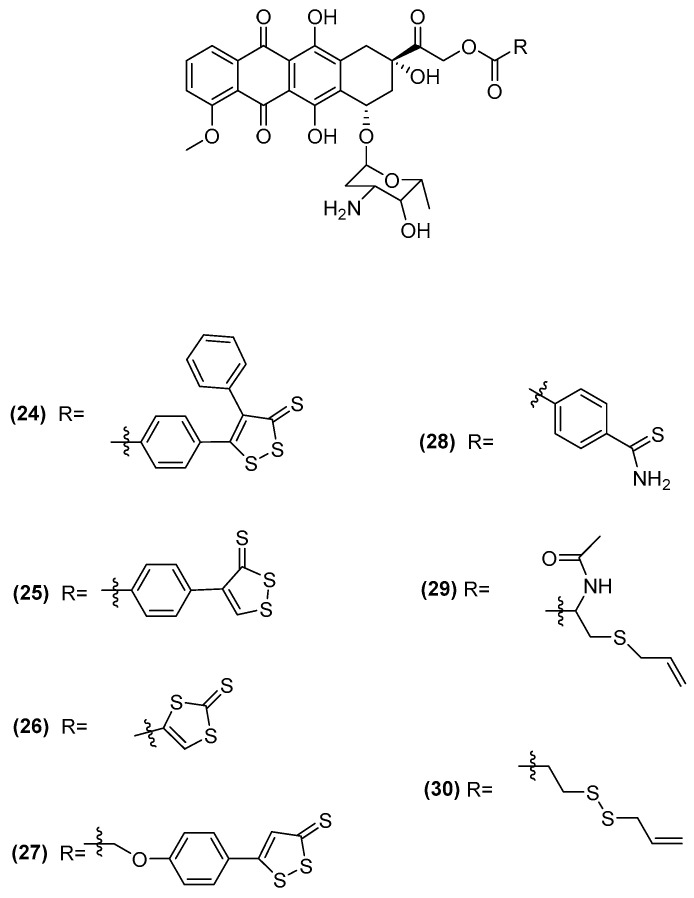
Structures of H_2_S-donating DOXOs.

Doxorubicin (DOXO) is a potent antineoplastic agent widely used in clinical practice, but its use is limited due to its cardiotoxicity and the rapid development of multidrug resistance. To address these issues, researchers have been developing H_2_S-releasing DOXOs (H_2_S-DOXOs) [66]. These new derivatives combine DOXO with H_2_S donor substructures (Figure 8) to reduce cardiotoxicity and combat DOXO-resistant tumor cells.

All H_2_S-DOXOs were evaluated on cardiac H9c2 cells, as well as DOXO-sensitive U-2OS osteosarcoma cells and their variants that exhibit varying levels of resistance to DOXO. The H_2_S-releasing moieties had a significant impact on the compounds’ biological activity. It was found that all H_2_S-DOXOs succeeded in diminishing the oxidative stress caused by the antibiotic in cardiomyocytes, with the majority also showing considerably lower toxicity compared to the original compound. In contrast to DOXO, the majority of these compounds are harmless to H9c2 cells at a concentration of 5 µM, showing promise for ongoing research and advancement [66].

Moreover, against sarcoma cell lines, all H_2_S-donating DOXOs demonstrated markedly stronger cytotoxic effects than the original compound [66].

#### 3.2.2. H_2_S-Donating Capsaicin Derivatives

Capsaicin (CAP), a well-known natural product found in hot peppers (Capsicum annuum L.), is a key TRPV1 agonist, offering health benefits. Studies on CAP have highlighted its pain-relief, cancer-fighting, anti-inflammatory, heart-protective, antioxidant, and weight-loss benefits, all of which are mediated by the activation of the TRPV1 receptor but are hindered by poor bioavailability and a tendency to irritate [67,68]. Despite attempts to alter CAP’s structure, no variants with a better pharmaceutical profile and less irritation have been successfully developed. 

**Figure 9:**
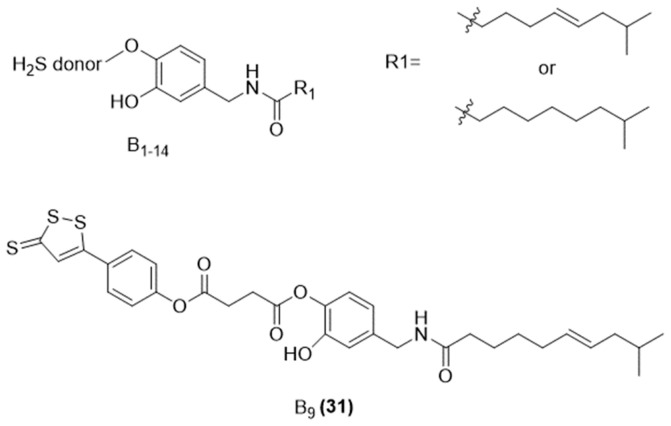
Chemical structures of H_2_S-donating capsaicin derivatives (B_1_-B_14_) and of the most interesting of the series (B_9_).

Consequently, aiming to enhance CAP’s effectiveness and reduce its irritant properties, a series of H_2_S-releasing CAP derivatives was developed by Qian and coworkers [69]. These compounds were synthesized by fusing capsaicin and dihydrocapsaicin with different hydrogen sulfide donors via an ester linkage to the C-4 position of CAP (Figure 9).

The new compounds underwent evaluation for their H_2_S releasing properties, analgesic and anticancer potential, and effect on gastric mucosa irritation. 

The results revealed that the addition of an H_2_S-releasing group significantly affected their biological actions. All the H_2_S-donating CAPs enhanced calcium influx, and most of them exhibited some level of pain-relief activity. Moreover, when tested against K562, Hela, and MCF-7 cancer cell lines, nearly all H_2_S CAP derivatives showed stronger cytotoxic effects compared to the parent compound CAP.

Notably, the evaluations revealed that compound 31 (Figure 9), which incorporates an ADT-OH as H_2_S donor, exhibited superior analgesic properties and stronger cytotoxicity against cancer cell lines compared to CAP. Additionally, compound 31 (Figure 9) demonstrated a significant reduction in rat gastric mucosa irritation caused by CAP. 

#### 3.2.3. H_2_S-Donating Metformin

Metformin, the most widely used oral antidiabetic drug, has found new potential uses, notably as an anti-cancer agent. 

Nowadays, there is a significant focus on both the fundamental and clinical research of metformin in the context of cancer. The key route for metformin’s anti-cancer effects involves the stimulation of the AMPK/mammalian target of rapamycin (mTOR) pathway, which is initiated by the suppression of complex I within the mitochondrial respiratory chain [70,71,72]. 

**Figure 10:**
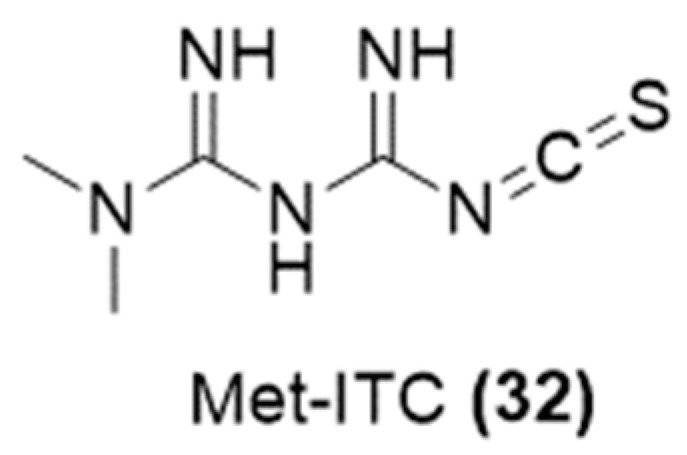
Chemical structure of H_2_S-donating metformin.

The urgent need to develop innovative strategies for cancer management and many studies confirming that H_2_S promotes anti-cancer effects in several tumor types [37,73] led Calderone and co-workers to develop a novel multi-target H_2_S-donating compound, called Metformine-ITC (Met-ITC) [74] (Figure 10), by incorporating an isothiocyanate moiety to metformin. 

Met-ITC demonstrated ability to release H_2_S both in cell-free assays and within cancer cells and greater efficacy in inhibiting cancer cell viability (e.g., AsPC-1, MIA PaCa-2, MCF-7) compared to metformin alone. It was less effective on non-tumorigenic cells (MCF 10-A). The presence of an H_2_S donor group, such as isothiocyanate function, gives the contribution to the anti-proliferative action by altering the cell cycle, promoting apoptosis, and suppressing the activity of histone deacetylases [74].

### 3.3. Pills of H_2_S-Donating Molecules as Cardioprotective Agents

In the cardiovascular system, numerous investigations have confirmed that H_2_S delivers advantageous and defensive actions, such as lowering blood pressure, enhancing vasodilation, mitigating atherosclerosis, and limiting endothelial dysfunction. Additionally, H_2_S is known for its antioxidant properties and its ability to promote the formation of new blood vessels. 

The significant progress in comprehending the biological functions of H_2_S and its mechanisms of action within the cardiovascular system [31] led the researcher to design innovative H_2_S-donating compounds.

#### 3.3.1. H_2_S-Donating Triphenylphosphonium

**Figure 11:**
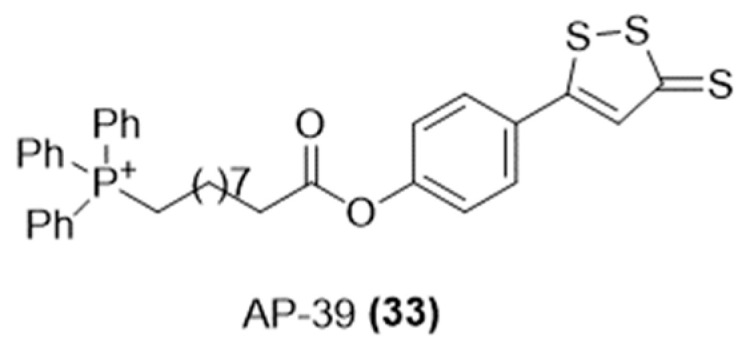
Chemical structure of H_2_S-donating AP-39.

Acknowledging the critical role of oxidative stress in endothelial mitochondrial dysfunction, which is a key factor in cardiovascular diseases, diabetic complications, inflammatory conditions, and various critical illness conditions, often linked to disruptions in H_2_S balance, Szabo et al. have developed a mitochondria-targeted H_2_S donor, known as AP-39 (Figure 11) [75]. 

This novel compound was synthesized through the combination of triphenylphosphonium and ADT-OH and then examined for its ability to release H_2_S and its potential therapeutic effects. AP-39 has been found to markedly reduce oxidative stress-induced toxicity and offer protection against severe cardiac arrest, as well as renal and myocardial ischemia/reperfusion (I/R) injuries, by blocking the mitochondrial permeability transition pore [76,77,78,79].

Moreover, Muscará and colleagues elucidated the mechanisms of action of AP39 on the vascular reactivity of mouse mesenteric arteries in vitro. Their studies revealed that the vasodilatory effect induced by AP-39 and its H_2_S-releasing component, ADT-OH, was significantly diminished following the removal of the endothelium, indicating a reliance on NO-cyclic guanosine monophosphate (cGMP) signaling and small-conductance calcium-activated potassium (SKCa) channel activation. These findings suggested that endogenous H_2_S is involved in AP-39’s mechanism of action, and the vasodilatory response was not altered by glibenclamide-induced KATP channel blockade [80].

More recently, novel studies indicated that AP-39 mitigates the cardiotoxic effects of DOXO by reducing oxidative stress, preventing cell death, and protecting against mitochondrial damage. This is achieved through the regulation of AMPK/uncoupling protein 2 (UCP2) expression [81].

#### 3.3.2. H_2_S-Donating Adenosine and Adenine Derivatives 

Considering the protective roles of adenosine and hydrogen sulfide in the cardiovascular system, particularly during ischemic/reperfusion events, Andreadou and colleagues have synthesized a new class of compounds that release hydrogen sulfide [82]. 

**Figure 12:**
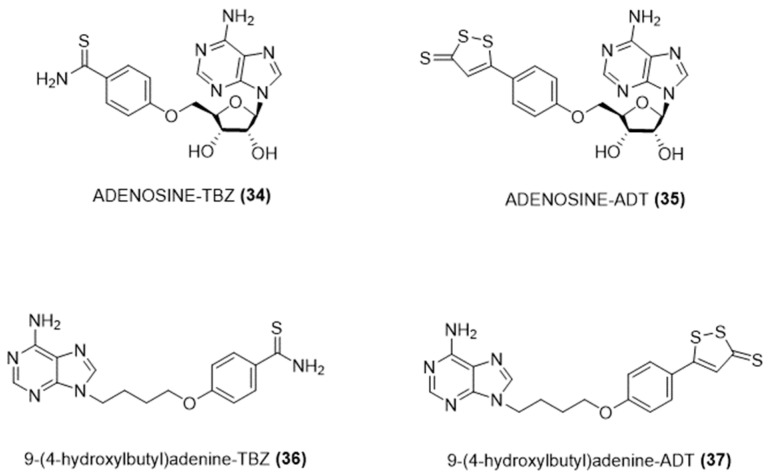
Chemical structures of H_2_S-donating adenosine derivatives.

Specifically, adenosine and the non-nucleoside analogue of adenine, 9-(4-hydroxybutyl)adenine, were chemically bonded with TBZ and ADT-OH through a stable ether linkage (Figure 12). 

These compounds were tested for their potential as cardioprotective agents both in vitro and in vivo. It has been shown that the novel hybrids were able to gradually release H_2_S and to significantly decrease the size of myocardial infarcts when used during myocardial ischemia [82]. 

#### 3.3.3. H_2_S-Donating Sildenafil

**Figure 13:**
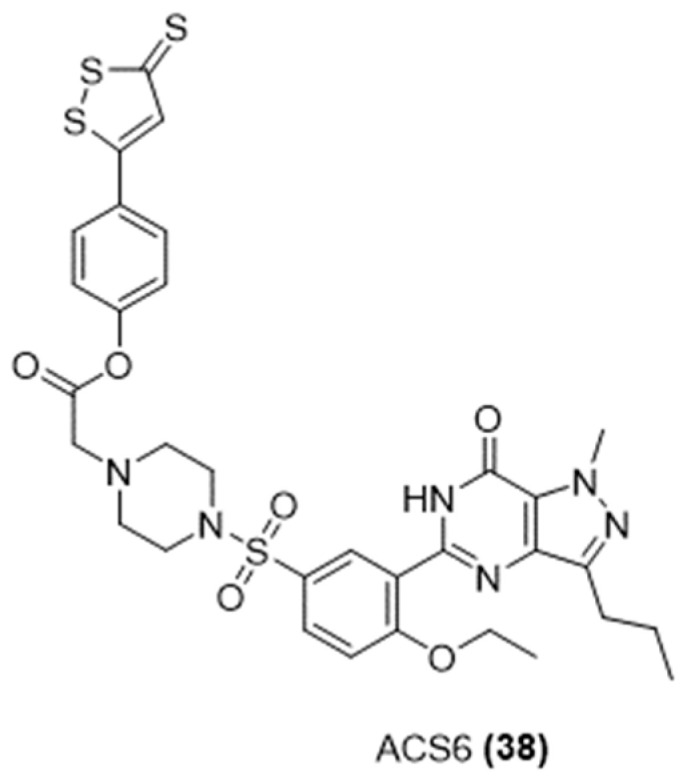
Chemical structure of H2S-donating sildenafil, ACS6.

Sildenafil, a phosphodiesterase-5 inhibitor (PDE5-I) commonly used in conditions like erectile dysfunction and pulmonary arterial hypertension, was combined with an H_2_S donor moiety, ADT-OH, obtaining a novel molecule named ACS6 (Figure 13) [83].

The new hybrid was found to be significantly stronger in relaxing the spongy smooth muscle compared to sildenafil, and it has been demonstrated that the greater effectiveness of ACS6 is due to its ability to gradually release hydrogen sulfide [84].

Moreover, ACS6 was more effective than NaHS solution at inhibiting the production of oxygen radicals in pulmonary artery endothelial cells. Unlike NaHS, which only operates through the cAMP/protein kinase A (PKA) pathway, ACS6 has the advantage of activating both the cAMP/PKA and cGMP/protein kinase G (PKG) pathways [84]. 

Furthermore, ACS6 has been shown to offer protection to PC12 cells by increasing the levels of paraoxonase-1 (PON-1), which helps counteract the neurotoxic effects induced by homocysteine and reduces oxidative stress [85,86]. 

### 3.4. Pills of H_2_S-Donating Molecules for Glaucoma Treatment

Glaucoma is a group of optic neuropathies characterized by retinal ganglion cell and axonal death, leading to irreversible vision loss. As glaucoma is one of the leading causes of blindness worldwide [87], researchers have been exploring novel therapeutic approaches to better manage this condition. 

Recent studies have shown that H_2_S has a protective effect against glaucoma-related damage [88,89,90,91,92]. 

In this context, new molecular hybrids that combine the action of antiglaucoma agents with H_2_S-releasing moieties have been designed and synthesized. These hybrids aim to provide a synergistic and enhanced therapy for glaucoma.

#### 3.4.1. H_2_S-Donating of Prostaglandin Analogs

**Figure 14:**
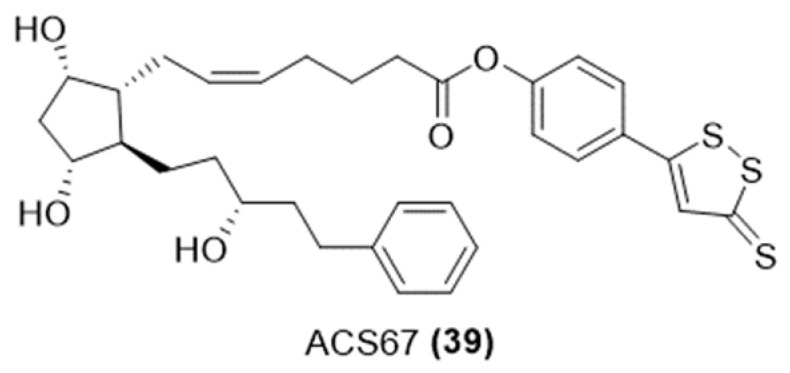
Chemical structure of H_2_S-donating Latanoprost.

ACS67 is a hydrogen sulfide-releasing derivative of latanoprost acid (Figure 14). It has been studied for its potential neuroprotective properties, particularly in the context of retinal health [93]. 

ACS67 has been shown to attenuate retinal ischemia and oxidative stress in RGC-5 cells in culture. This suggests that it could potentially be used to protect neurons in the retina from damage due to ischemia or oxidative stress [94]. The neuroprotective effects of ACS67 are attributed to its ability to release H₂S [93]. Studies have indicated that ACS67 can significantly blunt the negative effects of hydrogen peroxide (H₂O₂)-induced toxicity to RGC-5 cells, whereas Latanoprost alone did not. This highlights the additional benefit provided by the H₂S-releasing moiety of ACS67 [94]. While ACS67 was found to maintain the intraocular pressure (IOP) effectiveness associated with Latanoprost, its H₂S-releasing property added a neuroprotective dimension, making it a compound of interest for glaucoma treatment research.

#### 3.4.2. Other H_2_S-Donating Glaucoma Drugs 

**Figure 15:**
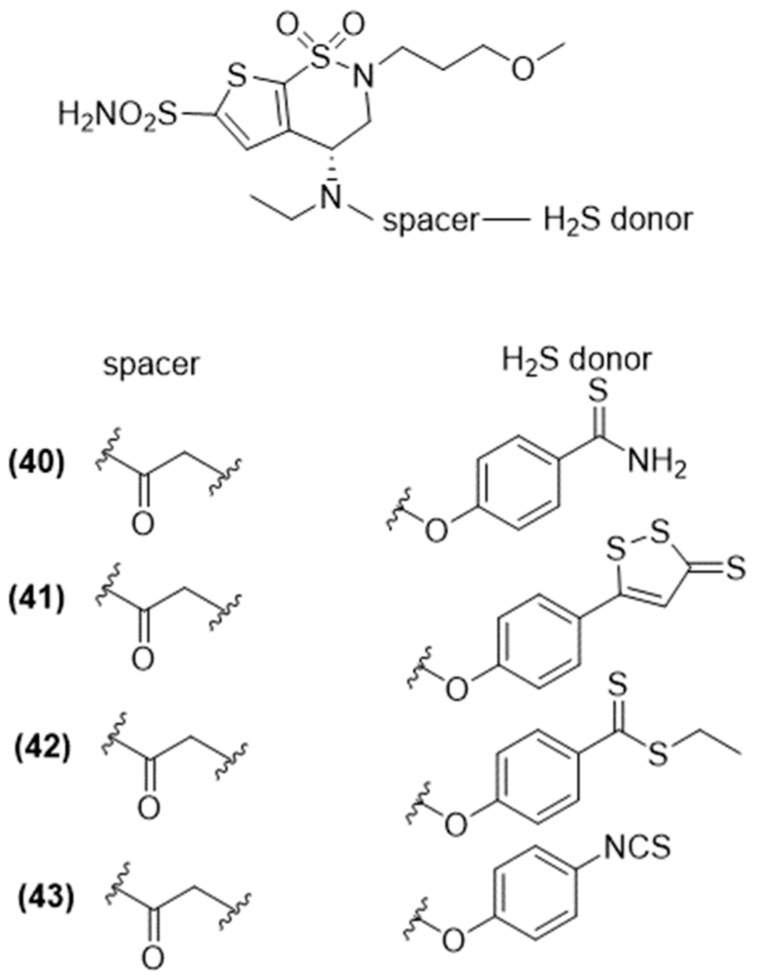
Chemical structures of H_2_S brinzolamide derivatives.

**Figure 16:**
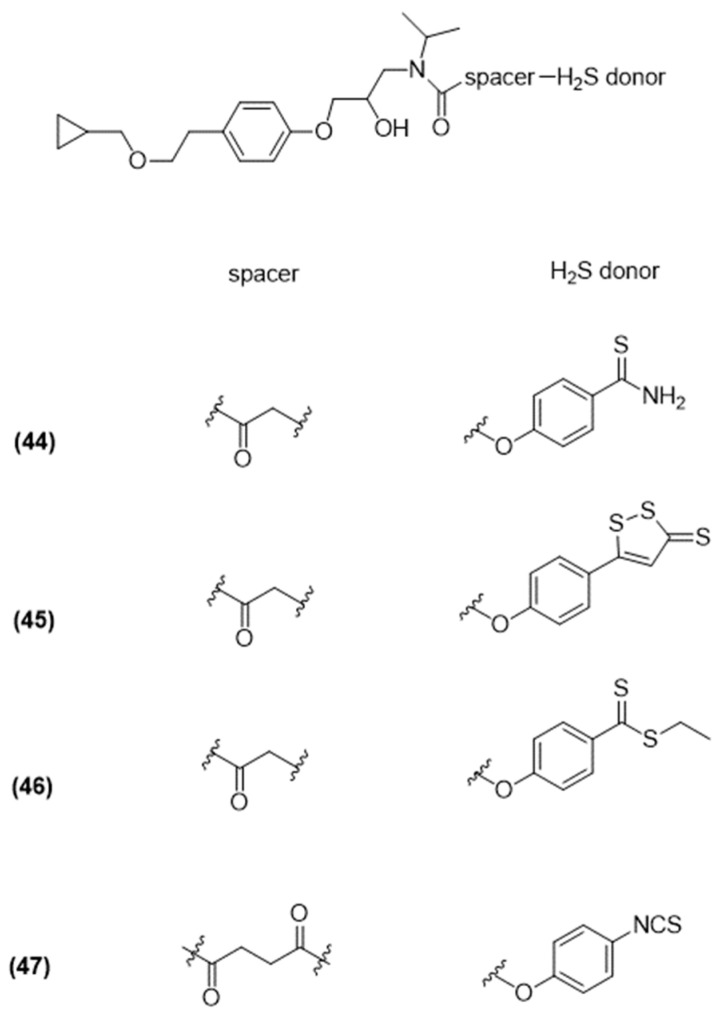
Chemical structures of H_2_S betaxolol derivatives.

**Figure 17:**
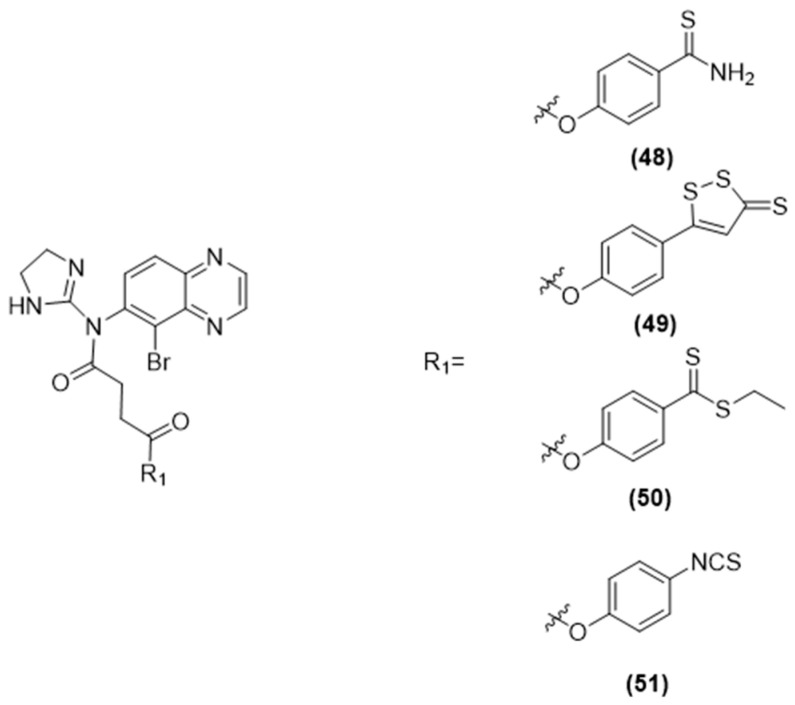
Chemical structures of H_2_S brimonidine derivatives.

More recently, Sparaco et al. selected other antiglaucoma drugs, including carbonic anhydrase inhibitors, such as brinzolamide (Figure 15), β-blockers, such as betaxolol (Figure 16), and α-adrenergic agonists, such as brimonidine (Figure 17), and coupled them with various H_2_S-releasing moieties [95].

The newly synthesized compounds were evaluated for their ability to release H_2_S using both amperometric and fluorometric methods.

The newly synthesized compounds’ ability to release H_2_S was confirmed by assessing an amperometric assay and in human primary corneal epithelial cells (HCEs) through spectrofluorometric analysis. Noteworthy compounds 42 (brinzolamide-HBTA), 43 (brinzolamide-HPI), and 51 (brimonidine-HPI) emerged as the most effective H_2_S-releasing hybrids in both aqueous solutions, in the presence of L-Cys, and within cells.

These initial findings support the concept of hybridization as an effective approach in drug development. Indeed, creating a new molecular entity by combining two or more drugs, identical or different, with or without a linker, aims to improve the effectiveness of the original drugs. Additionally, these results open up the possibility of pairing potent H_2_S donors like HBTA or HPI with proven intraocular pressure (IOP)-reducing medications, such as prostaglandin analogs, to develop innovative anti-glaucoma medications.

### 3.5. Pills of H_2_S-Donating Molecules for Neurodegenerative Diseases

Neurodegenerative diseases, like Alzheimer’s disease (AD), Huntington’s disease (HD), and Parkinson’s disease (PD) are persistent and progressive disorders. They result in neuronal loss, leading to cognitive and motor impairment. 

Unfortunately, to date there are no treatments that effectively manage symptoms or provide a cure for these diseases. 

Several studies reported the neuroprotective effects of the gaseous signaling molecule H_2_S, acting as a neuromodulator within the brain [96,97]. In particular, H_2_S interacts with both the NMDA receptor [98,99] and the α-amino-3-hydroxy-5-methyl-4-isoxazolepropionic acid (AMPA) receptor [100,101], influencing their functions. It shields nerve cells from oxidative harm by adjusting levels of GSH, managing KATP channels, and curtailing the generation of reactive oxygen species (ROS). Notably, the concentration of H_2_S and the activity of enzymes CBS and CSE are markedly reduced in the brains of individuals with neurodegenerative diseases [101,102,103].

Moreover, numerous investigations have highlighted the neuroprotective properties of H_2_S donors in both cell culture and animal models of neurodegeneration characterized by a decrease in H_2_S synthesis.

Consequently, therapeutic strategies based on H_2_S-donating hybrids for these conditions have been suggested.

#### 3.5.1. H_2_S-Donating Levodopa

Levodopa (L-DOPA) is currently an important drug in the treatment of Parkinson’s syndrome, but it only replenishes dopamine levels in the brain and does not inhibit the progression of the disease. 

Ongoing neuronal damage may be attributed to several factors, including oxidative stress, which encompasses the oxidation of L-DOPA and the neurotoxins produced by activated microglia and astrocytes.

**Figure 18:**
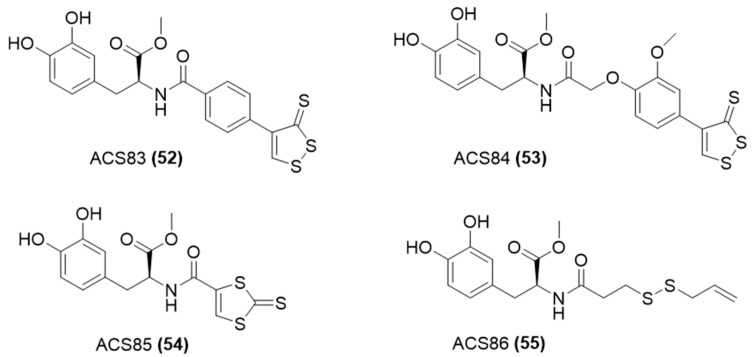
Chemical structures of H_2_S-donating DOPAs.

To mitigate these factors, a series of H_2_S-releasing derivatives, having both antioxidant and antiinflammatory properties, have been developed [104]. These compounds were obtained by combining L-DOPA with four H_2_S donors, such as ADT-OH derivatives and allyldisulfide (Figure 18).

H_2_S-donating DOPA hybrids were able to release both dopamine and H_2_S. They were also investigated in cell culture models and exhibited several beneficial effects, including antioxidant activity. Moreover, these hybrids reduced the levels of pro-inflammatory cytokines such as TNF-α and interleukin-6 (IL-6), as well as nitrite from stimulated human microglia, astrocytes, THP-1, and U373 cells. Additionally, they mitigated the toxicity of supernatants from the stimulated cells on SH-SY5Y cells [104].

Among the tested hybrids, ACS84 (4-(3-thioxo-3H-1,2-dithiol-4-yl)-benzoic acid, Figure 18) was particularly noteworthy. When injected into rats, ACS84 dissociated into L-DOPA and ADT-OH within one hour; in fact, both metabolites were detected in the brain.

In rat models of Parkinson’s disease, ACS84 improved motor deficits, reduced neuronal loss in the substantia nigra, and elevated dopamine levels in the striata [105].

Research has shown that ACS84 can avoid amyloid-induced neuronal cell damage through anti-inflammatory effects and protect mitochondria in p38- and JNK-mediated stress responses. Thus, ACS84 has the potential to treat neurodegenerative diseases [104,105,106].

#### 3.5.2. H_2_S Donating Memantine for Alzheimer’s Disease

AD is a progressive neurodegenerative disorder characterized by cognitive decline, memory loss, and other symptoms. Pathological hallmarks of AD include β-amyloid (Aβ) aggregation, τ-hyperphosphorylation, and loss of cholinergic neurons.

Despite significant research efforts, as for the other neurodegenerative diseases characterized by a complex etiology, discovering effective drugs for AD remains a major challenge. Currently, scientists are exploring the multitarget approach as a promising strategy to develop new medications for AD.

A clinically approved drug used in the treatment of AD, memantine, is an NMDA receptor antagonist that helps regulate glutamate activity in the brain, aiming to improve cognitive function and slow down disease progression. However, it has limitations, including its inability to halt the degenerative process [107]. 

**Figure 19:**
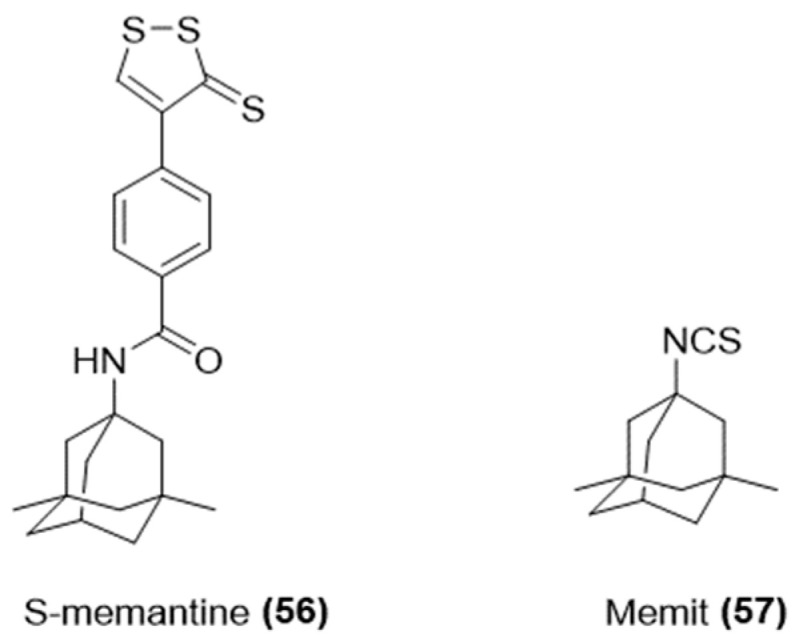
Chemical structures of H_2_S memantine derivatives.

Given the neuroprotective effects of the combination of exogenous H_2_S and NMDAR antagonism [108,109,110], H_2_S-donating memantine hybrids have been developed for this purpose. Indeed, Ichinose and co-workers conjugated the H_2_S donor ADT-OH to memantine, obtaining S-memantine (Figure 19) [111].

It has been shown that S-memantine, when compared to the parent ADT-OH molecule and Na_2_S, exhibited lower toxicity toward murine cortical neurons. Additionally, it enhanced cell viability in both SH-SY5Y and murine cortical neurons following oxygen-glucose deprivation and reoxygenation. Notably, post-treatment with S-memantine significantly improved survival rates and neurological outcomes in mice subjected to bilateral carotid artery occlusion/reperfusion. Remarkably, the effects of S-memantine surpassed those of Na_2_S, the parent ADT compound, and memantine alone [111].

More recently, Sestito et al. [112] developed another H_2_S-donating memantine compound by introducing an isothiocyanate group in place of the amine function of memantine (Figure 19). The novel chemical entity, referred to as “Memit,” has undergone in vitro testing to determine its pharmacological characteristics as the original drug. The studies have verified that Memit slowly generates H_2_S via a cysteine-dependent mechanism, resulting in the restoration of memantine. 

In neuron-like cells and microglia, Memit demonstrated various effects associated with H_2_S and memantine, such as offering protective benefits against neuronal inflammation and reducing ROS production. Memit has also been validated to lessen the accumulation of Aβ(1-42) and provide a protective effect against Aβ oligomers, which cause damage in human neurons and rat microglial cells. Moreover, this new molecule has been found to stimulate autophagy in U-87MG cells, which is disrupted in neurodegenerative conditions.

#### 3.5.3. H_2_S-Donating AChE Inhibitor for Alzheimer’s Disease

Tacrine (THA) was the first clinically approved acetylcholinesterase (AChE) inhibitor for AD treatment. Despite its clinical application, THA was withdrawn due to high hepatotoxicity. However, its high potency in AChE inhibition, low molecular weight, and simple structure make THA a promising scaffold for developing multitarget agents. 

**Figure 20:**
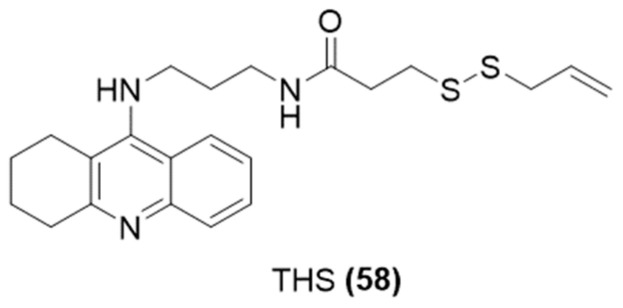
Chemical structure of H_2_S-donating tacrine.

In this context, considering the anti-inflammatory, hepatoprotective, and neuroprotective effects of hydrogen sulfide, Liu and co-workers have developed THA-based hybrids (THS, Figure 20) by combining THA with a derivative of the natural H_2_S-donating compound S-allylcysteine [113].

The obtained compound resulted in significant cognitive and locomotor activity improvements while addressing THA’s hepatotoxicity. The administration of THS successfully reduced the levels of AChE in both the serum and hippocampus of AD mice treated with AlCl_3_, showing effects similar to those of THA.

Furthermore, the compound THS diminished inflammation in the hippocampus, which was indicated by the lowered mRNA levels of inflammatory cytokines (TNF-α, IL-6, and IL-1β). Additionally, the hybrid compound enhanced the levels of H_2_S in the hippocampus, reduced inflammatory responses, and fostered better synaptic plasticity within the hippocampal region. Notably, this compound exhibited no signs of causing liver toxicity or inflammation, as determined by the levels of liver transaminases and inflammatory cytokines.

**Figure 21:**
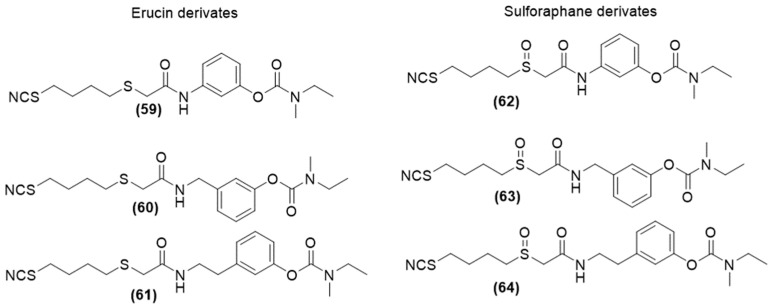
Chemical structures of H_2_S-donating rivastigmine derivatives.

Similarly, in 2019, Sesisto et al. selected another AChE inhibitor with brain-region selectivity and clinically approved drugs for AD [114], rivastigmine, and designed a new class of multitarget H_2_S-donating compounds (Figure 21) by combining it with two natural products, such as sulforaphane (SFN) and erucin (ERN), endowed both with antioxidant and neuroprotective effects [115]. 

This research study revealed that all newly synthesized hybrids demonstrated an in vitro H_2_S-donor profile and showed protective effects against LPS-induced inflammation in microglial cells. Additionally, these compounds have been observed to reduce NO production in cells stimulated with LPS and pre-treated with the hybrids.

The compounds also exhibited neuroprotective and antioxidant properties in SH-SY5Y neuronal cells. Compared to rivastigmine, which has no antioxidant activity, all hybrids significantly lowered ROS production triggered by pro-inflammatory stimuli. The new hybrids also decreased NO release in BV-2 microglial cells, unlike rivastigmine. This effect is largely attributed to the diverse mechanisms of action of the SFN and ERN groups and their ability to release H_2_S.

#### 3.5.4. H_2_S-Donating Melatonin for Neuro-Inflammation

Over the past years, melatonin has garnered considerable attention for its beneficial effects on the CNS, such as neuroprotective qualities, partly due to its strong antioxidant capabilities and its role as a radical scavenger. Owing to these properties, melatonin is being considered for the treatment of oxidative stress-related conditions, including neurodegenerative diseases (NDDs).

Given melatonin’s multifaceted nature and the promising neuroprotective effects seen with sulforaphane, it has been postulated that combining these compounds into a single molecule might yield synergistic, enhanced neuroprotective outcomes, potentially offering a new therapeutic approach for NDDs. 

**Figure 22:**
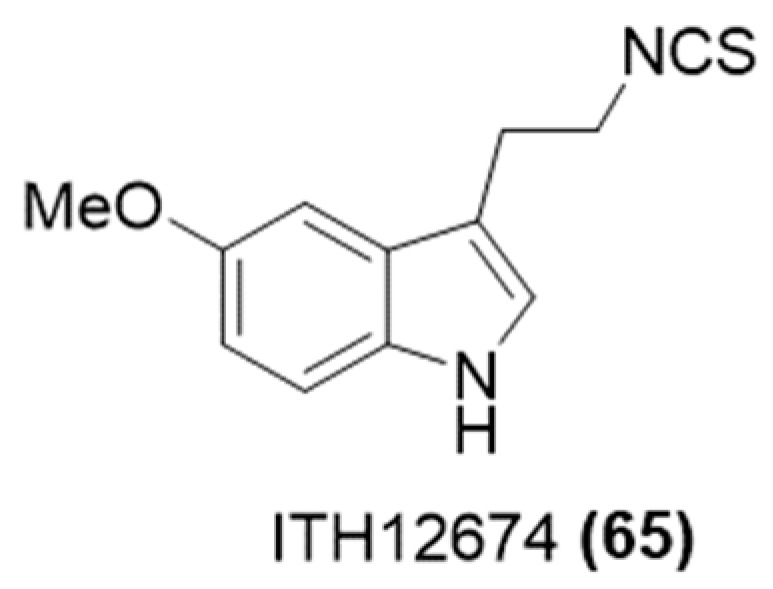
Chemical structure of H_2_S-donating melatonin.

In 2015, Leon and coworkers have thus designed a melatonin-sulforaphane hybrid known as ITH12674 (Figure 22) [116]. 

To evaluate the neuroprotective effects of the novel compound, various in vitro models simulating oxidative stress associated with neurodegenerative conditions and cerebral ischemia were utilized. This hybrid is engineered to interact with cysteines in Keap1, thereby releasing Nrf2. Indeed, it was able to interact with intracellular glutathione to form a powerful antioxidant that counteracts the excessive production of ROS and reactive nitrogen species (RNS). Owing to these synergistic actions, ITH12674 exhibited significant protection against oxidative stress. ITH12674 demonstrated a superior neuroprotective profile in comparison to melatonin and sulforaphane.

In 2020, in addition to its pharmacological assessment, the same research group also examined ITH12674 for its anti-inflammatory properties [117]. The obtained findings suggest that ITH12674 provided an anti-inflammatory response by inhibiting the Toll-like receptor 4 (TLR4) and NF-κB signaling pathways. This dual drug-prodrug action has led to an enhanced pharmacological profile, showing promise for treating NDDs. 

### 3.6. Pills of H_2_S-Donating Molecules for Osteoporosis

#### H_2_S-Donating Bisphosphonates

Bisphosphonates (BPs) stand as the primary treatment for osteoporosis, a complex metabolic bone disease characterized by low bone mass and deterioration of bone microarchitecture, leading to increased fragility and risk of fractures [118].

The pathophysiology of osteoporosis involves a disruption in the balance of bone formation and resorption, which is regulated by various genetic and environmental factors [118,119]. The strong bone affinity of BPs has led to their use in creating conjugates with drugs that either promote bone growth or prevent bone loss. 

**Figure 23:**
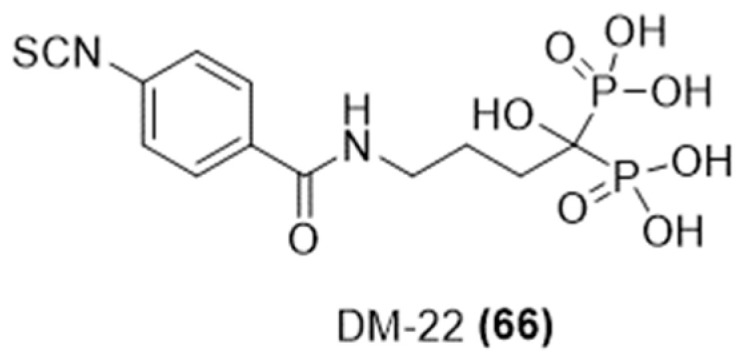
Chemical structure of H_2_S-donating alendronate.

Given recent findings indicating H_2_S as a significant molecule in bone metabolism, encouraging bone formation and hindering the differentiation of osteoclasts [120], Rapposelli et al. developed novel H_2_S-donating compound, DM-22 (Figure 23), derived from a combination of alendronate (AL), a bisphosphonate used for osteoporosis treatment, and an isothiocyanate group [121].

In vitro tests of DM-22 and AL assessed their impact on the survival and functionality of human osteoclasts and mesenchymal stromal cells during osteogenic differentiation. Amperometric analysis showed that DM-22 was able to release H_2_S gradually through a thiol-dependent mechanism. 

Notably, DM-22 markedly suppressed the differentiation and activity of human osteoclasts, preserving their viability. Unlike AL, DM-22 did not cause cytotoxic effects in human mesenchymal stromal cells. Therefore, DM-22 emerges as a promising candidate for a new generation of bone-anabolic drugs.

## 4. Conclusions and Future Perspectives

The development of H_2_S donating molecules is a dynamic field, aiming to create molecules that release H_2_S in a controlled manner. There are different types of H_2_S donors, including sulfide salts, garlic-derived sulfur compounds, and synthetic molecules with specific release mechanisms. These donors have potential therapeutic value, as they can modulate H_2_S levels in the body and may contribute to treatments for complex pathological conditions, such as inflammatory-based diseases, cancer, metabolic, cardiovascular, and neurodegenerative disorders.

In recent years, hybrid compounds that combine H_2_S donor moieties with native drugs have gained significant attention due to their potential therapeutic applications. 

One of the key advantages of the H_2_S-donating compounds is their ability to enhance the efficacy of native drugs by additional pharmacological actions attributed to H_2_S. By incorporating an H_2_S donor into a native drug, researchers have been able to simultaneously target multiple therapeutic pathways, resulting in improved treatment outcomes.

**Figure 24:**
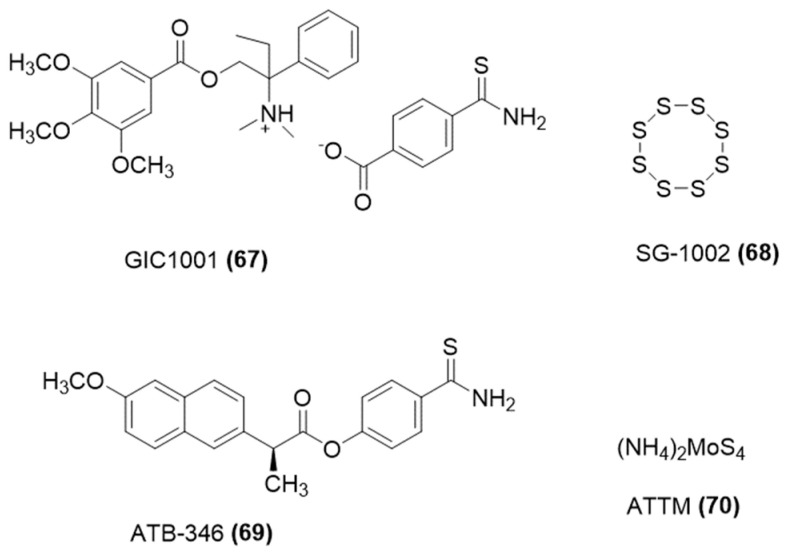
Chemical structures of H_2_S-donating molecules in clinical trials.

Notably, some developed H_2_S-releasing compounds are currently being evaluated in clinical trials for their therapeutic potential. Among them, noteworthy examples are reported in Figure 24 and include GIC-1001 from GIcare Pharma Inc., which is an H_2_S releasing trimebutine maleate salt, offering an alternative to traditional sedatives during colonoscopy procedures [122]; ATB-346 by Antibe Therapeutics, recognized for its anti-inflammatory properties [40] and recently included in phase II study targeting osteoarthritis-related pain; SG-1002 [123] by SulfaGENIX, which is under investigation (ClinicalTrials.gov NCT01989208) for its potential to elevate circulating levels of H_2_S and nitric oxide (NO) following heart failure and ammonium tetrathiomolybdate (ATTM) [124], which has undergone clinical trials for breast cancer treatment due to its ability to deplete copper.

Despite many advances in this field and great strides toward multi-target H_2_S donors, there are still some limitations to this application. 

A number of issues remain to be addressed, including the rapid release of H_2_S and fast metabolism in vivo, the capacity of H_2_S-related chemicals to target specifically their sites of action or to pass through the blood-brain barrier (BBB), and the optimization of laboratory techniques for measuring H_2_S levels in tissues or cells.

Therefore, while the development of hybrid compounds that combine H_2_S donors with native drugs represents a promising approach in drug discovery, further research is needed to optimize the design and delivery of these hybrid compounds for safe and effective clinical applications.

In conclusion, by summarizing the numerous H_2_S-donating molecules already developed and studied in different complex diseases, this review can enlighten researchers and lead them to develop new, increasingly effective, and promising hybrids.
